# Integration of mHealth Information and Communication Technologies Into the Clinical Settings of Hospitals in Sub-Saharan Africa: Qualitative Study

**DOI:** 10.2196/26358

**Published:** 2021-10-13

**Authors:** Oluwamayowa Oaikhena Ogundaini, Retha de la Harpe, Nyx McLean

**Affiliations:** 1 Department of Information Technology Faculty of Informatics and Design Cape Peninsula University of Technology Cape Town South Africa; 2 Graduate Centre for Management Faculty of Business and Management Services Cape Peninsula University of Technology Cape Town South Africa

**Keywords:** mHealth, health care professionals, co-design, hospitals, ActAD model, work activity, Sub-Saharan Africa, referrals, VULA mobile app, WhatsApp, mobile phone

## Abstract

**Background:**

There is a rapid uptake of mobile-enabled technologies in lower- and upper-middle–income countries because of its portability, ability to reduce mobility, and facilitation of communication. However, there is limited empirical evidence on the usefulness of mobile health (mHealth) information and communication technologies (ICTs) to address constraints associated with the work activities of health care professionals at points of care in hospital settings.

**Objective:**

This study aims to explore opportunities for integrating mHealth ICTs into the work activities of health care professionals at points of care in clinical settings of hospitals in Sub-Saharan Africa. Thus, the research question is, “How can mHealth ICTs be integrated into the work activities of health care professionals at points of care in hospital settings?”

**Methods:**

A qualitative approach was adopted to understand the work activities and points at which mHealth ICTs could be integrated to support health care professionals. The techniques of inquiry were semistructured interviews and co-design activities. These techniques were used to ensure the participation of frontline end users and determine how mHealth ICTs could be integrated into the point of care in hospital settings. Purposive and snowball sampling techniques were used to select tertiary hospitals and participants for this study from South Africa and Nigeria. A total of 19 participants, including physicians, nurses, and hospital managers, were engaged in the study. Ethical clearance was granted by the University research committee and the respective hospitals. The data collected were sorted and interpreted using thematic analysis and Activity Analysis and Development model.

**Results:**

The findings show that mHealth ICTs are suitable at points where health care professionals consult with patients in the hospital clinics, remote communication is needed, and management of referrals and report writing are required. It was inferred that mHealth ICTs could be negatively disruptive, and some participants perceived the use of mobile devices while engaging with patients as unprofessional. These findings were informed by the outcomes of the interplay between human attributes and technology capabilities during the transformation of the motives of work activity into the intended goal, which is enhanced service delivery.

**Conclusions:**

The opportunities to integrate mHealth ICTs into clinical settings depend on the inefficiencies of interaction moments experienced by health care professionals at points of care during patient consultation, remote communication, referrals, and report writing. Thus, the timeliness of mHealth ICTs to address constraints experienced by health care professionals during work activities should take into consideration the type of work activity and the contextual factors that may result in contradictions in relation to technology features. This study contributes toward the design of mHealth ICTs by industry vendors and its usability evaluation for the work activity outcomes of health care professionals.

## Introduction

### Background

The emergence of mobile health (mHealth) technologies originated from a need to provide support for health care professionals, workers, and patients to deliver and receive services efficiently at any time and anywhere. The concept of mHealth transcends beyond delocalized delivery of health care services. It includes the use of portable technologies (and sometimes enabled wireless communication) as a means of facilitating the processing and provision of timely information on the management of health care [1,2]. Thus, mHealth is referred to as the use of mobile technologies such as smartphones, tablets, and sensor-enabled hand-held devices for health-related purposes [3]. However, similar to other digital systems, there are challenges that hinder the effective use of mHealth information and communication technologies (ICTs) to support tasks within the work activities of health care professionals at points of care [4,5]. Health care professionals represent a skilled workforce that includes specialist physicians and nurses who carry out clinical work activities to provide care services at the point of care. Point of care refers to locations within clinical settings where health care professionals perform tasks that encompass the management of patients’ health and service delivery [6]. Thus, this paper explores how mHealth ICTs can be integrated into the work activities of health care professionals at the point of care in hospital settings to support the delivery of safe and quality care.

The existing literature has alluded to the claims that the outcome of technology-enabled work activities is influenced by the ability of health care professionals to make timely and informed decisions that ensure the quality of service delivery and, ultimately, improve patient well-being [7,8]. However, when adopted and designed without adequate consideration of the work activities executed by health care professionals, implemented health ICTs, including mHealth apps, are used partially or eventually discarded [9]. Although there is an increasing global penetration of mobile communication and devices in every other sector, such as commerce and banking, the application of mHealth ICTs by health care professionals at point of care in hospital settings is rarely explored, and the impact on service delivery is unclear [5,10,11].

The use of mHealth ICTs by health care professionals at the point of care has potential benefits. Owing to the information-intensive nature of health care services and the dependence of timely decision-making on up-to-date records, desktop computers may restrict the access, retrieval, and exchange of information to specific locations in hospital settings. Conversely, a major advantage of mHealth ICTs is that it enables real-time access to patient information and communication at different points of care and does not restrict the mobility of health care professionals [5]. It reduces time- and location-related constraints that may hinder health care professionals from quickly retrieving patient information for timely decision-making, especially where access to computer-based records is limited [12]. In case of emergencies, the use of mHealth ICTs provides a platform where health care professionals can easily verify clinical guidelines and related prescriptions in academic journals to make informed decisions and monitor patient vitals [6,13,14]. Ideally, mHealth ICTs are beneficial for addressing the shortcomings associated with timely retrieval of information and communication at point of care to ensure job and patient satisfaction after health care service delivery.

There are examples of initiatives in which mHealth ICTs have been developed to improve the care delivered by health care professionals to patients. Most of the initiatives are focused on specific public health–related health challenges for data collection by community- and home-based health care workers [15,16]. For example, MomConnect is built for affordable mobile handsets, with support for unstructured supplementary service data, SMS text messages, and voice communication. It offers messaging services that enable pregnant women to interact with health care providers through SMS text messages, asking questions, and submitting complaints to help improve their health as well as the health of their babies [17]. The Clinical Patient Administration Kit is a mobile-enabled electronic medical record tablet that assists clinicians in facilitating the tracking and reporting of treatment and outcomes in maternal and infant care services [18]. Clinical Patient Administration Kit improved the tasks performed by clinicians by eliminating repetition of tasks and records and providing timely care to patients at clinics.

It was drawn from the literature that treatments and well-being of patients have been extended out of the hospital to homes as a result of the access to and ownership of mobile technologies; however, there are limited studies on how tasks within work activities inform the use of mHealth ICTs by health care professionals at point of care in hospital settings. Other studies have investigated the attitudes and behaviors of health care professionals toward the acceptance and use of mHealth ICTs for its benefits [2,19-22]. In contrast to its benefits, there are concerns that mHealth ICTs could inhibit health care professionals when the nature of tasks performed within work activities is not adequately understood or considered.

Concerns have been raised by some authors regarding the unintended consequences associated with the use of mHealth ICTs and related applications within health care settings. In this paper, the authors refer to unintended consequences as contradictions that result from the tensions between sociotechnical interactions [23]. Unintended consequences can be either desirable or undesirable. For example, Watson et al [24] shared their views that despite its usefulness, WhatsApp (Facebook Inc) is not a secure communication channel for the exchange of patient clinical data because of weak encryption and the vulnerability of information during transmission. Therefore, it does not satisfy regulatory standards for use in health care [11]. In addition, Wallis et al [11] stated that mHealth ICTs are mostly not designed to interoperate with third-party apps or legacy systems. As a result, this causes fragmentation of data within the health care system. Other concerns highlighted by authors include the inhibition of workflow [7,25], time inefficiency [26], and influence on the patient-physician relationship [27].

### Activity Analysis and Development Model

This paper explores opportunities for integrating mHealth ICTs into the work activities of health care professionals at point of care using the Activity Analysis and Development (ActAD) model. The argument of this paper is that understanding the interaction between the elements of a work activity presents an opportunity for the integration of mHealth ICTs to enable the work activities of health care professionals at point of care and identify the contradictions that may influence unintended consequences associated with technology-enabled work activities.

The ActAD model is a theoretical approach used to describe the elements of a work activity and to gain insights on how their interactions inform the development of information systems [28]. A work activity comprises actors (individuals or groups) that use tools (means) to perform actions on an object that could be shared as informed by the motive of the activity and guided by a set of rules [29]. Ultimately, the motive of the activity is transformed into a goal or an intended outcome.

In the context of this study, work activities are defined as the set of actions performed by health care professionals according to their medical practice toward delivering health care services. Actions may include collection, access, and retrieval of information; sharing and exchange of information; communication between health care professionals; and use of the information to make informed decisions about patients’ health and well-being. The actions can be enabled by means of action that would be mHealth ICTs in the context of this study. The object of activity is the patient information or records, whereas the motive of the activity is to make an informed decision. Patient information could be a *shared object of networking* or *shared object of action,* depending on how it serves a group of health care professionals. Patient information enables health care professionals to make informed decisions on the state of well-being of patients to improve their health conditions. Ultimately, the motive of the activity is transformed into the intended goal or outcome of the activity.

Next, the research strategy and methods used to engage participants in exploring opportunities to integrate mHealth ICTs into the work activities of health care professionals at point of care are discussed, followed by data analysis results and interpretation of the research findings.

## Methods

### Qualitative Approach

The study was exploratory, as informed by the research objectives. The researchers adopted an interpretivist standpoint because of the subjective nature of socially constructed realities of different health care professionals who use (or do not use) mHealth ICTs for their work activities at the point of care. A qualitative strategy, including multiple techniques, specifically semistructured interviews and co-design activities, was used to engage the participants.

According to Myers and Newman [30], semistructured interviews enable the use of open-ended questions to gain insights and to afford opportunities for new areas to emerge for further exploration of a research problem. The use of co-design activities enables the users or potential users of a product or service to express their lived experiences and expectations visually, such that a researcher or designer can gain further insights into the use contexts [31].

The use of multiple techniques enabled researchers to draw inferences from the thoughts, experiences, and expectations of participants to understand the work activities of the health care service delivery process. More importantly, the techniques helped to understand the service interactions between health care professionals and technology-enabled work activities in a bid to identify opportunities to integrate mHealth ICTs into point of care hospital settings.

Participants were selected using purposive and snowball techniques. The 2 are nonrandom sampling techniques applied to select a sample size from a research population based on the biased judgment of the researcher in line with a research objective [32]. These techniques were applied to identify health care professionals who could provide a detailed account of daily clinical work activities and the tools used to enable specific actions within the process of care delivery. The snowball technique was adopted because of the difficulty in reaching available physicians who were willing to participate in the study because of their busy schedule. Thus, participants suggested some of their colleagues that could describe how technologies are currently being used and opportunities to integrate mHealth ICTs at point of care.

### Recruitment of Participants

#### Overview

A study by Abyaomi et al [22] on the factors that enable or inhibit the behavioral intention of physicians to use clinical informatics selected Nigeria and South Africa as leading economic powerhouses in Africa. According to a study on e-readiness of African countries conducted by Ifinedo [33], South Africa and Nigeria ranked within the top 5 countries in Sub-Saharan Africa with emerging technological innovation for a networked economy.

Health and the quality of health care service delivery contribute to the skills and well-being of human resources required for developing and sustaining economies. In this paper, the researchers considered Nigeria and South Africa to be 2 countries with comparable economic growth and are ideal for exploring the opportunities to integrate mHealth ICTs into the work activities of health care professionals to address the challenges that inhibit job satisfaction and quality health care service delivery.

#### Clinical Settings

The study was conducted in 2 tertiary hospitals, one in the Western Cape Province of South Africa and in the South Western geopolitical region of Nigeria because they provide specialized clinical care. The researchers purposively chose to investigate the workflow of health care professionals in clinical departments where activities are technology-enabled to ensure that the participants selected align with the aim of the study. For example, the authors considered clinical departments that deal with medical imaging and reporting where executing work activities involves the use of electronic systems and digitized records.

The 2 contexts comprised contrasting situations. In one of the tertiary hospitals, the physicians were using their smartphones and health ICTs, including a mobile app called VULA created by Dr William Mapham, whereas health care professionals in the other hospital largely used a paper-based system at points of care. In both tertiary hospitals, WhatsApp is used as a means of facilitating communication and sharing patient information.

WhatsApp is a consumer-oriented mobile app that can be installed to facilitate instant messaging in the form of text, pictures, audio, or video between two or multiple internet-enabled devices, including smartphones [34,35]. It offers a platform for health care professionals to exchange health-related information and communicate with each other or to follow up with patients on the status of their well-being [36]. WhatsApp could be interpreted as an improved version of the two-way pager used by health care professionals, which saves time, requires no computer, and limits communication barriers between colleagues.

Similar to WhatsApp, the VULA mobile app was designed to facilitate remote communication between health care professionals and workers as well as to manage referrals at points of care [37]. The VULA app allows health workers to seek advice and receive training from their more experienced colleagues or specialist physicians. The VULA app is an established referral platform used in the Western Cape Province of South Africa by clinical units, including orthopedics and ophthalmology, as a point of entry to facilitate the exchange of medical images and instant messaging related to real-time diagnostic and treatment consultation [38].

As described in Table 1, a total of 19 participants were engaged in this study and identified through purposive and snowball sampling techniques. In the tertiary hospital selected in South Africa, participants included 2 ophthalmology specialist physicians; 6 orthopedic specialist physicians and 3 nurses who manage trauma care wards, intensive care units and theater wards; and the deputy nursing manager of the hospital.

**Table 1:** Sampled participants and techniques of engagement.

Pseudocode for participants	Clinical department	Technique of engagement
oph1_H1^a^, oph2_H1	Ophthalmology	Face-to-face interviews at hospital H1 in the physicians’ library
ort1_H1, ort2_H1, ort3_H1, ort4_H1, ort5_H1, ort6_H1	Orthopedic	Face-to-face interviews and co-design activity at the physicians’ library
Deputy nursing manager (dnm_H1), trauma unit operational manager (tn_H1), theater operational manager (ton_H1), intensive care unit operational manager (icn_H1)	Nursing	Face-to-face interviews and co-design activities at the nursing board room
RR1_H2^b^, RR2_H2	Radiology	Face-to-face interviews at hospital H2 in the physicians’ consulting room and co-design activities
ortC_H2	Orthopedic	Face-to-face interviews and co-design activity at hospital H2 in the physicians’ consulting room
General surgeon (gs_H2); neurosurgeon (ns_H2)	Surgery	Face-to-face interviews and co-design activity at hospital H2 in the physicians’ consulting room
Assistant director of nursing services (adn_H2); Orthopedic nurse (on_H2)	Nursing	Face-to-face interviews and co-design activity at hospital H2 in the physicians’ consulting room

^a^H1: South African hospital.

^b^H2: Nigerian hospital.

In the Nigerian tertiary hospital, participants were 2 radiologist physicians, 1 orthopedic nurse, 1 assistant nursing director in radiology, 1 orthopedic consultant, 1 neurosurgery consultant, and 1 general surgery consultant. A common drawback in the 2 contexts is that physicians were mostly unable to honor appointments because of their busy schedules. This is a common phenomenon reported in a study by Kabanda and Rother [39].

#### Data Collection Process

Data were collected from both tertiary hospitals from September 2018-January 2019. Hospital managers were the first point of contact at the hospitals. The hospital managers directed the heads of clinical departments that deal with medical imaging and reporting to assist experienced physicians and nurses who were interested and willing to participate in the study. The interested participants were contacted by email, and appointments were set up based on their convenience and subject to their availability.

Two sets of data were collected from the participants. The interviews and co-design activities took place within the hospitals’ premises as convenient for each participant. Each interview lasted an average of 40 minutes, whereas each of the co-design activities lasted an average of 60 minutes. The participants were allowed time to express themselves as they could, whereas the sessions were recorded with a portable voice recorder.

During each co-design activity, one of the researchers assumed the role of a facilitator to incorporate their knowledge of the issues of investigation and the research design procedure to encourage participants’ active participation. The motivation for using a co-design activity was to comply with ethical concerns and avoid any disruptions that are associated with directly observing health care professionals’ workflow in real time.

In preparation for the co-design activities, a set of paper cut-out graphic representations of physicians, nurses, and the tools that portray health care work activities, as indicated in the literature, were provided to participants. Cut-out representations of actors and tools provide an opportunity for participants to visually express and map their actions and experiences to researchers [40]. In addition, large pieces of paper, pencils, erasers, and stickers were provided to health care professionals as writing materials to illustrate the directions of their workflow.

The participants performed three tasks during the coactivity sessions. The first task was for participants to visually illustrate the start of their daily work activities, how they perform their tasks, and the tools used, if any. Subsequently, the participants used the cut-outs to represent themselves, as actors, on a large piece of paper using the stickers, thereby giving practical descriptions of the actions undertaken at the point of care. The outcome of the first task was a visual illustration of the workflow or user journeys of health care professionals from the first encounter with a patient until the patient is discharged, transferred, or deceased.

For the second task, the researcher used visual illustrations generated by participants to identify the touchpoints within the user journeys. In this paper, touchpoints are described as contact points where a network of human actors and objects interact to produce or influence an outcome [41]. By identifying the touchpoints, the researcher was able to determine the interaction moments of tasks performed by health care professionals during work activities. The interaction moments could be human-to-technology, human-to-human, or machine-to-human actions.

The researcher then probed using open-ended questions to determine whether there were any challenges experienced by health care professionals at any point or while using health ICTs within the visual illustration generated. The outcome of this task produced the challenges, unintended consequences, and the resulting effects attributed to the use of health ICTs by health care professionals.

For the third task, the outcomes of the second task were used to facilitate a discussion between the researcher and participants on how mHealth ICTs could be integrated into their work activities at the point of care. The outcome of the third task presented an opportunity to identify the characteristics of the expected features that a fit-for-purpose technology could have to enable health care professionals’ work activities. This marked the end of the co-design activities.

Ultimately, the data collected from the semistructured interviews were used to inform and complement the coactivity sessions used to engage with physicians and nurses. By doing so, the researcher was able to validate what health care professionals said against their actions. Both techniques enabled the researcher to identify opportunities to integrate mHealth ICTs into the work activities of health care professionals at the point of care in a hospital setting.

### Ethical Considerations

To ensure that the research was executed in a manner that guarantees safety and harm-free procedures to the participants, the researchers, and the environment, an ethical clearance was obtained from all relevant authorities. The authorities include, first, the University’s research ethics committee, then the researcher applied to the Western Cape Provincial department of health, South Africa, and the tertiary hospital in Nigeria. Permission was granted on the condition that the researchers adhered to the delineation and safety measures highlighted in the ethics clearance application.

An information sheet was provided to each of the individual participants explaining that participation was voluntary, and consent could be withdrawn if they felt that the questions being asked were uncomfortable. In other words, participation was not incentivized. The participants signed a consent form that allowed interview and co-design sessions to be recorded, and they were aware that the information collected would be used in a confidential manner and responses would not be misconstrued. For security purposes, the data obtained from the participants were stowed away in a password-protected folder on a secured desktop computer at all times.

### Data Analysis Process

Thematic analysis was used to sort and organize qualitative data. The process of thematic analysis enabled the researcher to determine the frequency of attributes to present the findings [42]. First, the recorded data collected from participants were transcribed from audio to verbatim text to allow the researcher to easily categorize the data into themes. To group the data into themes, the researcher used descriptive codes to tag the emerging attributes, which are words or phrases that imply the issues being investigated, and then categorize the codes into themes [43]. The processes through which attributes emerge are identified were conceptualization and operationalization.

Conceptualization refers to defining the keywords of a research question or objective, whereas operationalization is used to identify the attributes that characterize the keywords through a process of coding [32]. The coding process involved assigning a descriptive word to identify points where mHealth ICTs were being used and could be integrated into the work activities of health care professionals at the point of care. Ultimately, this enabled the researcher to sort and organize the data collected from the interviews and the co-design activity transcripts. The themes were informed by the elements of the ActAD model, as shown in Textbox 1.

**Themes and findings generated from the categorizing of transcript codes.**

**Research objective**

- Explore opportunities for integrating mobile health (mHealth) information and communication technologies into the work activities of health care professionals at point of care

**Themes**

- Actions: Work activities are patient consultations, remote referrals, and formulating treatment plans
- Tools: mobile apps—VULA and WhatsApp
- Outcomes of the work activities: this attributed to the use of mobile apps for work activities
- Unintended consequences associated with the use of mobile apps for work activities
- Transformation process: alignment of mHealth apps to work activities of health care professionals

**Findings**

- The nature of work activities is routinized and require health care professionals to have timely access to updated patient information and to reduce their mobility between point of care.
- VULA and WhatsApp are mobile apps used by physicians to facilitate communication, exchange patient information, and enable remote consultations.
- Other hospital information systems are used to facilitate the retrieval of patient records (objects of activity) and booking of clinical examinations to diagnose and make informed decisions (motive of work activity).
- VULA has simplified the process of referrals and reduced unnecessary referrals.
- VULA enables accountability of the referral process.
- VULA and WhatsApp have improved how physicians communicate and collaborate with each other remotely.
- VULA referral notifications interrupt physicians during patient consultation multiple times.
- Responding to VULA referral notifications is time consuming.
- Use of mobile apps by physicians at point of care might be perceived by patients as unprofessional.
- Use of mobile apps at point of care could enable distractions from the work activities.
- VULA mobile app is suitable for referrals, retrieval, and the exchange of health-related information.
- VULA mobile app requires a push notification of referrals to health care professionals that are available while on call.
- mHealth app suitable for patient consultation may include a voice recorder with speech recognition to capture and transcribe verbal communication.
- Other features may include high-resolution cameras and web-enabled and instant messaging to reduce interaction time at point of care.

## Results

### Overview

The results were presented using the elements of the ActAD model to deductively describe how mHealth ICTs could be integrated into the work activities of health care professionals, as informed by the interaction moments of tasks and outcomes. Insights were drawn from the interviews and co-design activities used to engage with all participants. The analysis showed that patient consultation, referral management, treatment planning, and report writing were the main work activities executed by health care professionals.

### Consultation With Patients

#### Overview

The physicians indicated that their first point of contact with patients in the hospital was by the outpatient clinics where they had to access and retrieve patient records or engage with their patients to collect a history record.

During this activity, physicians use hospital information systems on computer desktops, mobile phones, and paper. The interaction moments during patient consultation include verbal communication, taking notes, retrieving patient records, and requesting clinical examination:

In our clinics, for all patients that are seen notes are also made by hand and those notes as well as all referrals goes into a patient’s folder. All those notes are sent to the scanning department. And all notes get scanned into our ECM. All those notes eventually do become available on a computer.

oph1_H1

On the basis of the accounts of specialist physicians, it is evident that work activities at point of care are supported using technology. The electronic content manager is used to store patients’ records electronically. Another physician explained as follows:

VULA is a smartphone based app where doctors and healthcare professionals, that includes more than just doctors, can have direct communication with on-call doctor or Orthopaedic person, to ask for advice and or refer patients by a means of a list of questions as well as photos of x-rays that can be sent through to us.

ort2_H1

In hospital H2, physicians and nurses stated that they used their mobile phones at point of care but did not use any forms of hospital information systems or mHealth ICTs. However, they were aware of the benefits associated with technology-enabled work activities at point of care:

During call hours we make use of our mobile phones and some social media apps: WhatsApp, Snapchat. Now, this we use mostly to send images, radiological images and even some laboratory reports. So, a lot of times if a resident is reviewing a patient somewhere and is conversing over the phone, a lot of times I tell them to send images on my phone.

ortC_H2

At the clinic, doctors and the nurses make use of paper to take the record of the patients and when they are through...to assess the patient, then all the records are documented.

on_H2

It can be inferred that when mobile phone apps are used at point of care, it serves as a quicker means to facilitate communication and easier information exchange between health care professionals. The use of mobile phones provides an opportunity for physicians and nurses to capture information digitally, thereby reducing the amount of time and papers used manually. Thus, there is an opportunity to improve the outcome of tasks performed during consultation with patients or when seeking medical advice remotely.

#### Outcome of Consultation Enabled by mHealth ICT

Health care professionals are able to communicate directly with each other to view necessary medical images remotely and to save time on patient diagnosis and treatment decision-making. Ultimately, VULA mobile apps have reduced both the time and cost implications associated with clinical decision-making by health care professionals. Physicians explained as follows:

When referring a patient with VULA application the referring doctor must give a lot of important information about the patient to us that include a photo of the eye and then we can have a conversation with them to get a better idea of the problem. This way of referring is much more comprehensive that a telephonic referral, and we can get a better idea of the problem.

oph2_H1

VULA makes it easier to make clinical decisions without seeing patients personally. It’s often difficult to determine if a patient can be seen as an outpatient or do they need urgent referral, and using the VULA app with clinical pictures helps with this decision making.

ort4_H1

Technology-enabled referrals afford physicians the opportunity to request detailed information from their colleagues about patients before making informed decisions, without the need for a face-to-face consultation. One of the physicians in hospital H2 alluded to the importance of technology-enabled work activities:

Digitising would really help a lot, then I will not have to be going on all around. That will help a lot to reduce unnecessary waiting time. Many times, we are at the clinic, we have to be waiting for folders to arrive before we start.

ns_H2

The responses show that VULA app as an example of mHealth ICTs is useful for the work of health care professionals. The usefulness is evident in how mobile devices can reduce the time taken to access and retrieve patient records remotely at any time or at different point of care. mHealth ICTs provide a platform for conducting extended investigations through the use of instant messaging. Despite the benefits associated with mHealth apps, there is a concern about VULA notifications being disruptive to work activities while physicians are attending to patients at point of care during consultations.

#### Disruption by Mobile Referral Notifications

Physicians in hospital H1 revealed that during patient consultations, they received VULA referral notifications on their mobile phones. The referral notifications are received multiple times and from multiple sources, and they cause distractions to their work activity at point of care:

During the day it actually interferes and it slows you down massively. Definitely because you have a lot of patients that you need to see, you need to answer the phone; you need to answer your bleeps and then you also get VULA referrals. And some of them—I would say half of them, aren’t emergencies and those people want an answer now because they’ve got a patient sitting in front of them but I’ve got 50 patients sitting outside.

oph1_H1

It is evident that there are contradictions that result from a lack of understanding of how the tasks within work activities of health care professionals are carried out at the point of care. Thus, the design of VULA mobile app influences how mHealth ICTs are experienced at the point of care:

I feel that it seems unprofessional to constantly be looking at your phone screen whilst consulting patients.

ort5_H1

According to ortC_H2, mobile devices are used to access academic journals while teaching:

These days we tend to use our mobile device in clinic, but really we should have a laptop...if you see patients most times, you have residents and students. You can check up journals’ abstracts to compare and to show them some images and pictures.

ortC_H2

The physician further mentioned that mobile devices are seldom used in wards to facilitate teaching of medical students “because they believe it tends to be a distraction.” The response indicates that the nature of work activities should be adequately considered before designing mHealth ICTs relevant to tasks performed at points of care.

### Referral Management

The use of mHealth ICTs enables accountability by tracking the referral source and receiving comprehensive feedback on consultations. As established earlier, the VULA app enables health care professionals to remotely communicate and access real-time information (including text and images). The motive of activity in the instance of patient referrals enabled by VULA app as a means of action is transformed into the intended goal of activity by health care professionals:

There is a massive advantage. So, firstly is that I know who is sending me the referrals, I know where the referral is coming from. One thing we have picked up with regards to where referral is coming from is sometimes the name of the referring healthcare worker comes up but also their location. But that location is what they put in. It’s not a location that is picked up by the smartphone...so it doesn’t give you a location at that time but it’s a location is filled in.

ort2_H1

At hospital H2, one of the specialist physicians indicated challenges with the use of paper folders:

Many times, you don’t find many folders when you see patients. For example, that shows that a lot of them are misplaced somewhere or maybe didn’t find them properly at the record...some claim they are taken by one doctor or another...

ns_H2

Accountability is attributed to the use of the VULA appl. However, there are instances of inconsistency with geotagged locations and locations filled by the referring health care professional. This raises concerns when health care providers attempt to locate the exact institution of the referring health care personnel and the particular area where a patient is likely to be referred from. In addition, where paper folders are being used at point of care, there are no adequate accountability measures to track location or movement.

### Collaborative Treatment Planning

Health care professionals, including specialist physicians and nurses, use their personal phones to make calls and use WhatsApp as a means of communication and coordination to plan care delivery. Furthermore, having a personal mobile phone helps health care professionals locate their colleagues, especially when the official communication medium (switchboard bleep) takes a longer time or stops being functional:

Within the department, everyone’s got a work group on WhatsApp, so that’s to communicate about meetings. And then sometimes you just pick up your own phone...if switchboard takes too long to speak to someone...unless you don’t have the doctor’s number then you have to wait for switchboard. I use my phone actually; we do use our phones a lot.

oph1_H1

In hospital H2, one physician explained as follows:

There is disjointed management of patients, where a doctor comes and does his job and the nurse is not there; and then a physiotherapist comes and everyone sees the patient disjointedly. There is no team approach to patient care.

ortC_H2

The convenience of having a means of action such as mobile technology and an mHealth ICTs app facilitates quicker communication between health care professionals and eliminates any restrictions associated with mobility between point of care in a hospital setting. Thus, the communication gap during collaborative care coordination is bridged.

### Report Writing and Administering of Care

The responses indicate that nursing activities can be categorized into administering of care to patients and report writing while assisting physicians in facilitating clinical examination:

Some documentation gets lost during movement of patients. It is time consuming because whatever procedure is completed, the nurse got to come down and do the right thing afterwards. The paper, it’s illegible many times due to the fact that peoples’ handwriting differ.

dnm_H1

The nursing staff indicated that report writing was a major part of their nursing activities and required a lot of paper, as they were required to accompany patients to and from the different point of care. A shared sentiment among nursing staff who participated in the interview and co-design activity is that continuous use of paper can be addressed by introducing mHealth ICTs designed to facilitate report writing and documentation of patient care on-the-move:

From the points of assessment—casualty around the ward, from the point of assessment we need a gadget that can take patients’ parameters. It will be better if it’s mobile, since this would allow easier movement and should be interconnected to all wards, clinics so that at a click, it sends the information concerning the patient to other nurses at the next points-of-care.

ons_H2

The findings drawn from the *Results* section indicate that the opportunities to use a means of action such as VULA and WhatsApp by health care professionals at point of care depend on how the technology enables the motive for performing an activity is eventually transformed into the intended goal of an activity. Otherwise, contradictions as a result of a lack of mHealth ICTs *fit* could mediate how the motive of activity results into an unintended outcome.

## Discussion

### Principal Findings

On the basis of the accounts of most health care professionals, the use of mHealth ICTs for work-related activities has mostly yielded and would ensure positive outcomes at the organizational and individual levels. For example, physicians in particular use hospital information systems to facilitate easier access to, and retrieval of, patient records electronically during patient visits. In this study, the use of VULA mobile app for referrals simplified the referral process and reduced the unnecessary referrals to the hospital. The findings support the arguments by O’Connor et al [5] that the use of mHealth ICTs can enhance the task performance of health care professionals at point of care. In terms of communication and the writing of clinical notes, physicians can use mobile smart devices to record their conversations to obtain a comprehensive history from patients [44]. Ultimately, the relevance of mHealth ICTs in clinical settings is evident in information access, exchange, and communication.

WhatsApp was indicated as a means used by most physicians to communicate and exchange information. According to mHealth studies by Wallis et al [11] and Ganasegeran et al [45], health care professionals tend to communicate easily through WhatsApp by sharing patient information, including pictures, and seeking advice. However, the authors highlighted that the use of WhatsApp has potential risks to patient confidentiality because there are no built-in security measures and consent is a requirement for the exchange of patient information.

WhatsApp features could enable physicians to give adequate attention to patients while engaging in verbal communication during consultation. In this study, the voice-to-text feature on WhatsApp seemed to be ideal to assist health care professionals in digitally capturing patient history because it is readily available on smartphones. However, there are limitations to the use of the WhatsApp voice-to-text feature that makes the app inadequate to enable interaction moments during patient consultations.

One of the limitations of WhatsApp voice-to-text feature is that it only coherently transcribes verbal communication in English. This puts a patient who speaks an indigenous language at a disadvantage. In addition, there is no time stamp distinction between the voices of the physician and a patient, which would result in a cluttered clinical history note. Therefore, an mHealth voice-to-text and speech recognition app is essential for consultation but should ensure that there is a clear distinction between voices and during transcription from audio to text when used in the clinical context of health care service delivery.

In the wards, where the availability of desktop computers is limited and fixed, smartphones can be used to access electronically stored patient records. This requires health data records to be integrated across all digital platforms. In addition, physicians tend to use their smartphones to organize their work activities. An investigation by Greer et al [46] established that health care professionals use smartphones as a means to access or offer answers to medical questions and cross-reference evidence-based treatment medication from web-based academic sources in clinical settings. Similarly, O’Connor et al [5] investigated the impact of mHealth on the provision of care as perceived by physicians. The authors suggested that mHealth ICTs should be easily adaptable to perform the tasks within the work routine of health care professionals in hospital settings. Thus, the integration of health ICTs plays an important role in enhancing the decision-making of health care professionals in diagnosis and treatment processes to ensure the delivery of quality and safe care in clinical settings.

According to Cresswell et al [47], when systems inadequately meet the preferences of the intended end users to achieve an anticipated outcome, they are likely to adopt alternate means or discard the technology perceived as inadequate. Despite benefits attributed to the perceived usefulness and ease of use of health ICTs, there are unintended consequences experienced by health care professionals. The physicians expressed that the frustration caused by referral notifications received from the VULA mobile app can be disruptive multiple times while consulting with patients and during off-peak hours away from the hospital.

The unintended consequences could be attributed to social and technical mediators that inhibit the quality of care provided at point of care. This causes a delay in the time it takes to complete a consultation to the extent that some physicians feel uncomfortable with attending to VULA referrals. Gagnon et al [7] identified the compatibility of mHealth technologies with tasks as one of the several factors perceived to enable or inhibit the workflow of health care professionals. According to Yahya [26], time constraints and perceived reactions of patients to the use of mHealth ICTs by physicians could inhibit the optimal use of mHealth ICTs. The use of mHealth ICTs in clinical settings is more efficient when location is an inhibiting factor and tasks require minimal to no distractions during work activities, provided there are sufficient wireless bandwidth connections.

The nursing staff in tertiary hospitals is heavily saddled with the responsibility of patient administration, including admission, discharge, and transfer. As the amount of paper becomes cumbersome depending on the extent of a patient’s journey, a mobile device is ideal to support report writing and other information management tasks when nurses move from one point of care to the other. The task benefits or unintended consequences experienced by health care professionals while using mHealth ICTs to enable interaction moments influence the degree of its suitability, job, and patient satisfaction [48]. Otherwise, the mHealth ICTs would be partially used or even discarded when health care professionals are not satisfied with the values attributed to use, and ultimately, health care service delivery continues to be impaired by a lack of adequate technology integration [20,47,48].

These findings contribute to pertinent considerations when making informed decisions to design, develop, or purchase mHealth ICTs. This study encourages ICT and health care professionals to work in a transdisciplinary team during the design phase of health technologies. This saves time and costs involved in facilitating training for health care professionals in the pilot and postimplementation phases.

### Limitations and Future Research

There were difficulties and delays in setting up interviews with health care professionals in tertiary hospitals. The difficulties were because of the busy schedules of health care professionals in public hospitals. Hence, this restricted the data collection process to clinical departments that provide medical imaging and reporting care only. In the South African context, health care professionals used health ICTs, including mHealth, whereas in the Nigerian context, there were no health ICTs used. The authors adopted a pragmatic approach to include unstructured open-ended questions.

The results of this study are not generalizable because the investigation considered only 2 tertiary hospitals in 2 countries in Sub-Saharan Africa, and the data are subjective. However, the methodology can be replicated, and the lessons learned in these contexts can be compared with similar hospital settings. Future studies should consider conducting extensive usability tests of mHealth ICTs applicable to the work environment of health care professionals and standardization of information sharing across different information systems in hospitals. For example, the time efficiency and usability of mHealth ICTs during patient consultation require speech recognition, medical semantics (context sensitivity), and transcription app. Additional features such as a time stamp are also needed to distinguish between voices during transcription from audio to text. These mHealth ICT mechanisms would reduce the amount of time expended by health care professionals at point of care, given the number of patients and referral consultations attended to on a daily basis.

### Conclusions and Contribution

The findings of this study show that mHealth ICTs could be used during clinic consultations where physicians need to capture verbal communication with patients and observe their body language. The VULA mobile app is useful for managing referrals and information exchange remotely in a timely manner, except for its interruptions when physicians are busy with face-to-face consultations and other urgent activities. Owing to the mobile nature of nurses, mHealth ICTs could ease information capture, retrieval, and report writing between different point of care, particularly where computer access is limited and to reduce the amount of paper used for patient care administration in hospital settings. WhatsApp enables easier communication through instant messaging and offers a means to collaborate between professionals; however, in health care contexts, it does not guarantee the privacy of patients’ health information.

The opportunities identified can be considered by local health policy makers and ICT vendors when designing mHealth ICTs to enable the work activities of health care professionals at point of care in hospital settings, especially in Sub-Saharan Africa. This paper provides a possible direction for stakeholders in the public health system of Sub-Saharan Africa that have recognized and adopted the use of technology as a driver to address time and location constraints that impede service delivery.

This study applied the ActAD model as a lens to understand opportunities to integrate mHealth ICTs into the work activities of health care professionals in hospital settings. The authors established that interaction moments of tasks performed by health care professionals during complex work activities are essential to identify relevant mHealth ICTs for different point of care. In addition, the transformation of the purpose of a work activity into the intended outcome is mediated by the interplay between attributes of human agency and contradictions that emerge from the contextual conditions and the technical characteristics.

## Abbreviations

ActAD: Activity Analysis and Development
ICT: information and communication technology
mHealth: mobile health
